# Phenic Acid as an Antiseptic

**Published:** 1866-01

**Authors:** 


PHENIC ACID AS AN ANTISEPTIC.—Experiments have been made to show that the miasmata from putrefying substances may be rendered harmless by means of phenic acid, since it prevents or puts an end to spontaneous fermentation. A piece of perfectly fresh meat was placed within about two yards of one in a state of putrefaction. In eight days it was found merely to have begun to dry, the phenic acid had destroyed the miasmata, so that they were not transmitted by the air. It has been concluded, from these and similar results, that phenic acid is a powerful anti-pestilential agent, and it is considered extremely useful in places where cholera or other infectious maladies prevail.

